# Mid Pleistocene foraminiferal mass extinction coupled with phytoplankton evolution

**DOI:** 10.1038/ncomms11970

**Published:** 2016-06-17

**Authors:** Sev Kender, Erin L. McClymont, Aurora C. Elmore, Dario Emanuele, Melanie J. Leng, Henry Elderfield

**Affiliations:** 1 Centre for Environmental Geochemistry, School of Geography, University of Nottingham, Nottingham NG7 2RD, UK; 2 British Geological Survey, Keyworth, Nottingham NG12 5GG, UK; 3 Department of Geography, Durham University, Durham DH1 3LE, UK; 4 Department of Earth Sciences, University of Cambridge, Cambridge CB2 3EQ, UK; 5 DST—Università del Sannio, Via dei Mulini 59a, Benevento 82100, Italy

## Abstract

Understanding the interaction between climate and biotic evolution is crucial for deciphering the sensitivity of life. An enigmatic mass extinction occurred in the deep oceans during the Mid Pleistocene, with a loss of over 100 species (20%) of sea floor calcareous foraminifera. An evolutionarily conservative group, benthic foraminifera often comprise >50% of eukaryote biomass on the deep-ocean floor. Here we test extinction hypotheses (temperature, corrosiveness and productivity) in the Tasman Sea, using geochemistry and micropalaeontology, and find evidence from several globally distributed sites that the extinction was caused by a change in phytoplankton food source. Coccolithophore evolution may have enhanced the seasonal ‘bloom' nature of primary productivity and fundamentally shifted it towards a more intra-annually variable state at ∼0.8 Ma. Our results highlight intra-annual variability as a potential new consideration for Mid Pleistocene global biogeochemical climate models, and imply that deep-sea biota may be sensitive to future changes in productivity.

## Abstract (web summary)

The cause of a 20% decline in sea floor calcareous foraminifera species during the Mid-Pleistocene remains enigmatic. Here, the authors present new geochemical evidence, from the Tasman Sea that supports a change in phytoplankton food source as the primary driver.

## Graphical abstract

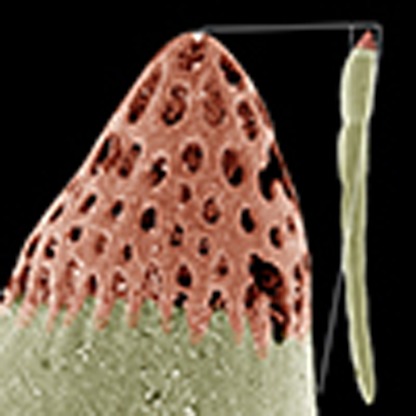

High-resolution continuous records, such as those found in the marine realm, provide an outstanding opportunity to pair fossil occurrences with geochemical environmental proxies to examine the possible causes of mass extinctions. The Mid Pleistocene transition (MPT, ∼1.2–0.6 Ma) was characterized by global cooling, glacial stage lengthening, changing ocean circulation and evolution of terrestrial and marine biota^1^,^2^,^3^. During the MPT, a stepwise extinction of over 100 species of deep-water benthic foraminifera occurred, targeted to a specific morphological group (largely the elongated ‘stilostomellids' with ornamented apertures, referred to here as the ‘extinction group') within just three families^4^,^5^,^6^,^7^. Benthic foraminifera are an important group often comprising >50% of the total benthic eukaryote biomass on the deep-ocean floor^8^. The cause of this last mass extinction, their only significant extinction in the last 15 Myr, is still unknown^6^,^7^,^9^,^10^,^11^. The deep ocean is the largest and most stable habitat on Earth^10^, and the only other two mass extinctions of benthic foraminifera in the Cenozoic occurred over the Palaeocene–Eocene and Eocene–Oligocene boundaries^10^. Understanding the ecological sensitivity of the deep sea is important as it is a habitat increasingly under stress^12^, and may be sensitive to current and future changes in productivity^13^. This last mass extinction in benthic foraminifera, completed by ∼0.8–0.6 Ma, has been extensively studied and shown to be global in extent, although slightly diachronous and apparently stepwise during glacials^4^,^7^,^11^,^14^. Higher latitude localities (above ∼60^o^) have not been studied in detail^7^, but Bering Sea^15^ and Kerguelen Plateau Southern Ocean^7^ foraminiferal assemblages show very low abundances of the extinction group before the MPT. A lack of living representatives has hampered our understanding of their ecological preferences and therefore the cause of the extinction. Leading hypotheses include the following: changing deep-water physical properties, such as cooling and increased oxygenation^6^,^7^,^10^,^14^; a change in productivity or extinction of a particular phytoplankton food source^7^,^11^; and increased ‘seasonality' or annual irregularity of organic carbon export^9^,^10^,^11^. Although bottom-water temperature, oxygenation and corrosiveness have been challenged as the cause, partly because of a global uniform change being unlikely^7^,^10^, these properties are yet to be directly tested with environmental parameters reconstructed alongside extinction group data^7^.

In this study, we address these hypotheses by examining the first records of extinction group abundance generated together with proxies for bottom-water temperature, carbonate saturation (Δ[CO_3_^2−^], related to corrosiveness), sea-surface temperature (SST) and productivity. We also generated records of phytoplankton assemblages from two sites to compare with a published South Pacific record^11^. Here we provide direct evidence from benthic foraminiferal Mg/Ca which disproves the hypothesis that changing bottom-water temperature controlled foraminiferal decline^6^. However, we do find compelling micropalaeontological evidence for a change in phytoplankton food source as the cause of the extinction. We show that a global dominance of the nannoplankton ‘small' *Gephyrocapsa* occurred at ∼0.8 Ma, coinciding with the largest decline in benthic foraminifera. We conclude that small *Gephyrocapsa* evolution may have enhanced intra-annual variability of primary productivity and carbon flux reaching much of the mid-low latitude ocean floor where the extinction was centred, and that the extinction group may have been unable to cope with long periods of seasonally reduced organic carbon flux during parts of the year.

## Results

### Benthic extinction in the Tasman Sea

**Figure 1:**
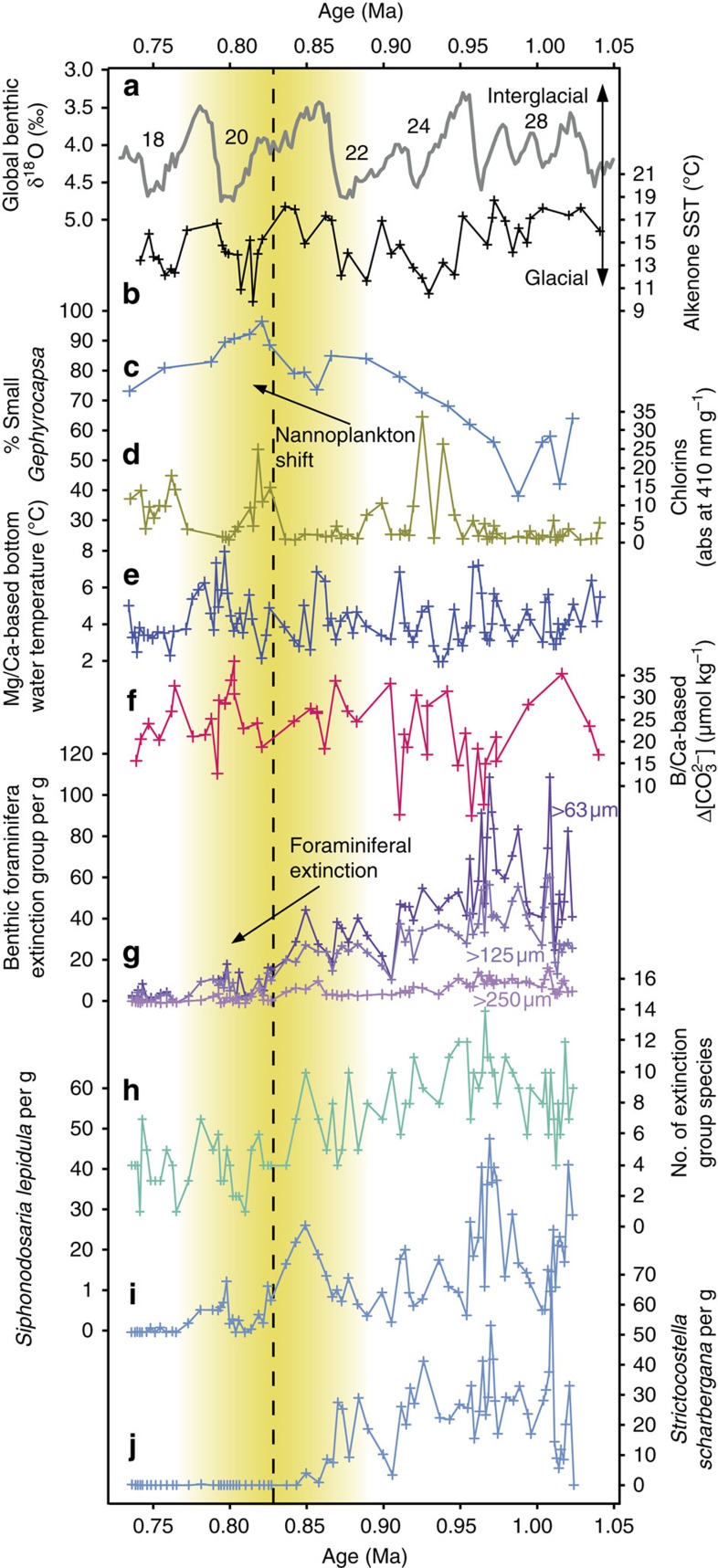
Reconstructed environmental proxies for Tasman Sea DSDP Site 593. (**a**) Global benthic foraminifera δ^18^O composite^52^ showing colder glacial (positive) and interglacial cycles. (**b**) Sea-surface temperature reconstructed from alkenones. (**c**) Small *Gephyrocapsa* as a % of total nannofossil assemblage. (**d**) Concentration of chlorin pigments used here as a proxy for photosynthetic material related to primary productivity^24^. (**e**) Bottom-water temperature reconstructed from benthic foraminiferal *U. peregrina* Mg/Ca ratios. (**f**) Bottom-water Δ[CO_3_^2−^] reconstructed from benthic foraminiferal *C. wuellerstorfi* B/Ca ratios. (**g**–**j**) Abundance per g and number of species of benthic foraminifera from the extinction group. Note that none of the environmental proxies (**d**–**f**) follows abundance of the foraminiferal extinction group (**g**–**j**). The vertical yellow bar indicates the interval over which the foraminiferal extinction occurs, and the vertical dashed line indicates where small *Gephyrocapsa* dominates the assemblage.

We reconstructed environmental parameters using a wide range of proxies at Deep Sea Drilling Project (DSDP) Site 593 over the period ∼0.75–1 Ma, covering the benthic extinction (Fig. 1). Site 593 is situated in 1,063 m of water on the Challenger Plateau of the Tasman Sea (SW Pacific Ocean, Supplementary Fig. 1), lying to the north of the modern Subtropical Front, which is a complex zone delineated by large gradients in SST and salinity^16^. SSTs in the Tasman Sea are considered to be sensitive to glacial–interglacial displacement of the Subtropical Front^7^,^17^. Site 593 is bathed by Antarctic Intermediate Water, which is broadly characterized by low salinity (34.3–34.5 PSU), low temperatures (3.5–10 °C) and high dissolved oxygen (200–250 μmoles kg^−1^; refs 18, 19).

Previous low-resolution (∼22 kyr) benthic foraminiferal data from Site 593 (ref. 5) indicated that the extinction occurred between 0.8 and 0.9 Ma. Our higher-resolution (∼3.5 kyr) results indicate the extinction group declined in the Tasman Sea, irrespective of size fraction, throughout the study interval with an initial overall reduction in abundance at ∼0.95 Ma, and a major decline towards very low abundance at ∼0.83 Ma (Fig. 1g). Our data are consistent with other studies that conclude that the architecture of the extinction is captured in all size fractions^7^, and suggest that it was not associated with an early shortening of the life cycle, which might be apparent with an increased proportion of small specimens. Sites below ∼1 km water depth typically have the highest extinction group diversity^7^, and species richness at Site 593 is relatively low at ∼10–12 species per ∼10-cc sample (Fig. 1h). Site 593 is dominated by *Strictocostella scharbergana* and *Siphonodosaria lepidula*, which decline abruptly at 0.85 and 0.8 Ma, respectively (Fig. 1i,j). Different species are dominant in extinction assemblages in the Pacific, Atlantic and Mediterranean, although they are morphologically (and thus possibly ecologically) related and also became extinct during the MPT^7^.

### Bottom-water temperature and corrosiveness

**Figure 2:**
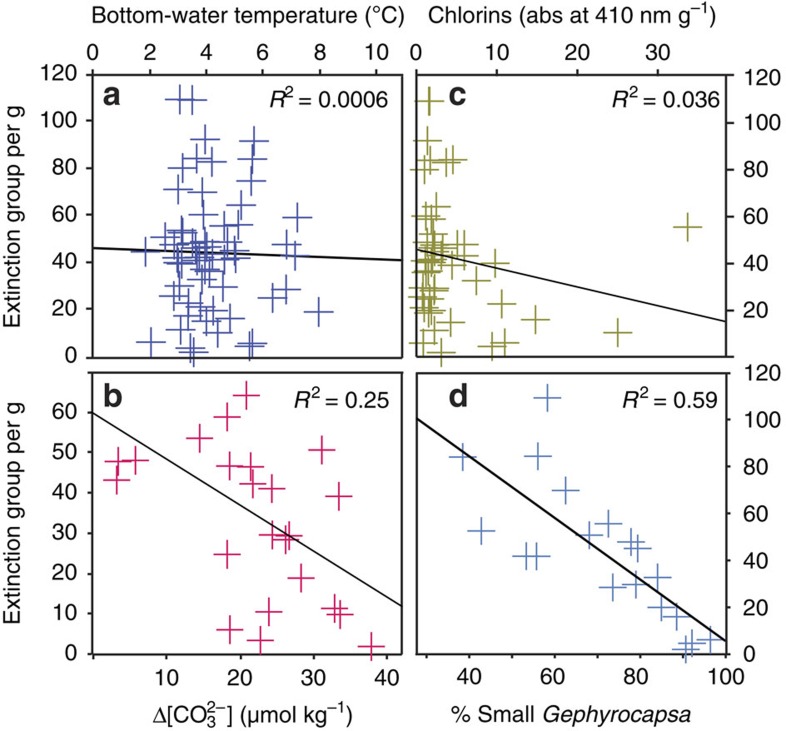
Foraminiferal extinction group against other parameters for the pre-extinction interval ∼0.8–1 Ma. (**a**) Bottom-water temperature derived from foraminiferal Mg/Ca, (**b**) bottom-water Δ[CO_3_^2−^] derived from foraminiferal B/Ca, (**c**) chlorin P410 from bulk sediment and (**d**) % small *Gephyrocapsa.*

Decreased intermediate/deep-water temperature is a hypothesized cause of the extinction^6^, possibly due to increased oxygenation and its impact on an inferred microbial food source^6^,^10^,^14^. Bottom-water temperature at intermediate-depth Site 593, reconstructed from Mg/Ca of infaunal *Uvigerina peregrina* (see Methods), ranges from ∼3 to 8 °C (modern temperature is 4.5 °C), with warmer interglacials (Fig. 1e). Seawater absolute magnesium (17 Ma residence time) and calcium (1 Ma residence time) concentrations would have been slightly different during the Mid Pleistocene^20^, thus having an impact on the accuracy of the temperature estimates based on modern calibration (possibly by up to ∼1 °C), although the overall trends will be unchanged. There is no apparent correlation between bottom-water temperature and faunal abundances during the pre-extinction period before ∼0.83 Ma (Fig. 2a), nor any secular change over the extinction itself, and we conclude that the benthic extinction at Site 593 was not caused by temperature changes. Increased bottom-water corrosiveness is another physical property that has been proposed to have an impact on benthic foraminifera^21^,^22^. The living position of the extinction group is unknown, but has been inferred as infaunal^7^,^9^,^11^. If this were the case, pore water [CO_3_^2−^] might be more relevant regarding the extinction, although pore waters have lower [CO_3_^2−^] and changes in bottom-water [CO_3_^2−^] would have influenced pore water values (in addition to other factors such as organic carbon flux and sedimentation rate). Bottom-water Δ[CO_3_^2−^] at Site 593, reconstructed from B/Ca of epifaunal *Cibicidoides wuellerstorfi* (see Methods), shows relatively low values ranging from 10 to 25 μmol kg^−1^, with highest values recorded during cooler glacials and some potential negative outliers before 0.9 Ma (Fig. 1f). Similar to Mg/Ca, the absolute B/Ca of seawater would have been slightly different from modern values, thus potentially having an impact on the absolute values of our calculated Δ[CO_3_^2−^] even though the overall trends should be considered accurate^23^. Since all the values are oversaturated with respect to *in situ* [CO_3_^2−^] (that is, all have positive Δ[CO_3_^2−^] values), and the extinction group abundance does not co-vary with Δ[CO_3_^2−^] during the pre-extinction interval (Fig. 2b), we conclude that the extinction group was tolerant to values in this range and could not have become globally extinct because of increased bottom-water corrosiveness.

### Carbon flux

Benthic foraminifera are influenced by several ecological factors, which include bottom-water oxygenation, bottom-water sediment heterogeneity and hydrodynamics, temperature, corrosiveness and organic carbon type and flux^21^,^22^. However, above the lysocline (below which calcite dissolution occurs), typical open-ocean benthic ecology is primarily affected by the organic carbon flux—which is related to primary productivity (quantity, type and duration) and remineralization of particulate organic carbon as it is transported to depth^10^. A change in organic carbon export, linked to primary productivity, has been hypothesized as an alternative cause of the extinction^7^,^9^,^10^. SST at Site 593 (Fig. 1b), reconstructed using the alkenone proxy U^K^_37_′ (see Methods), shows significant variability over the study interval, ranging from 10 to 18 °C, with cooler temperatures during glacials after ∼0.95 Ma. Since modern SSTs in the Tasman Sea are tightly coupled to the position of the productive Subtropical Front, we anticipate that shifts to Subtropical Front position might have influenced the organic matter flux. Sedimentary chlorin (Fig. 1d) is derived from photosynthetic material and specifically from chlorophyll pigments^24^. At this distal oceanic location they are likely to have originated from a proximal phytoplankton source, although a terrestrial contribution cannot be discounted by this study. Chlorin concentrations are typically higher during colder glacials at 0.75, 0.82 and 0.93 Ma, possibly indicating enhanced productivity^24^ at times of Subtropical Front northward migration (cooler SSTs). However, despite significant variations, there is no prior correlation (Fig. 2c) nor secular change in chlorin concentration (and by extension phytoplankton carbon flux), which could account for the extinction in benthic foraminifera focused at ∼0.83 Ma (Fig. 1).

### Phytoplankton food source

The extinction group therefore appears to have been relatively tolerant to variations in physical water properties and potentially overall organic carbon flux changes at Site 593 before the ∼0.83 Ma decline. Changing organic carbon *source* is another possible extinction mechanism^7^,^9^,^10^,^11^. The extinction group probably lived infaunally, according to their elongated morphology and lower shell δ^13^C than epifaunal foraminifera^7^,^9^,^11^, and preferred relatively high organic carbon flux at upper-abyssal to mid-bathyal depths; their global abundance reflects this distribution^7^. The specialized architectural function of extinction group apertures has been discussed at length^11^ but remains unknown, and may have helped direct pseudopodial flow for detritus feeding^10^,^11^, perhaps leaving them sensitive to a change in organic carbon supply.

**Figure 3:**
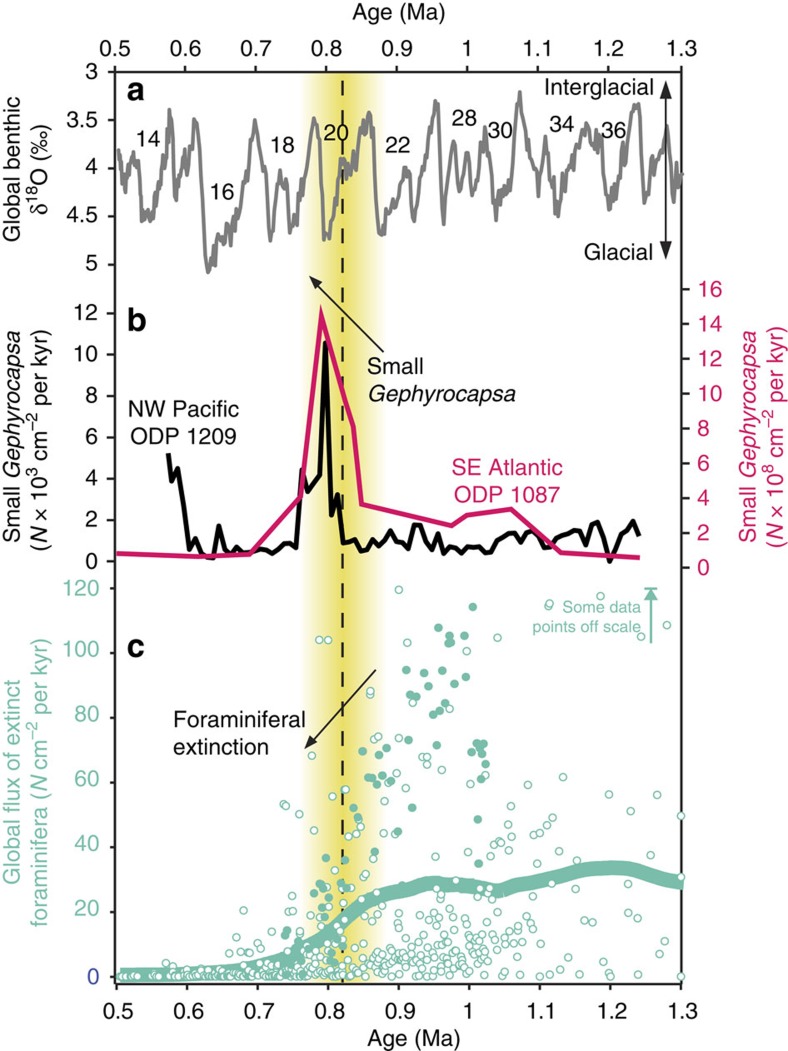
Nannoplankton assemblages compared with extinct benthic foraminifera over the Mid Pleistocene. (**a**) Global deep-sea δ^18^O composite^52^. (**b**) Accumulation rate of small *Gephyrocapsa* at Ocean Drilling Program (ODP) Site 1087 (ref. 26) and ODP Site 1209 (ref. 27). (**c**) Flux (accumulation rate) of extinct foraminifera from 15 global sites compiled by ref. 7 (open symbols), and including new data from this study (solid symbols), with a 0.2-pt LOESS smoothing spline (bold). Some data points are off the scale; smoothed line takes into account all data. Note how the peak in small *Gephyrocapsa* dominance at ∼0.8 Ma occurs in the NW Pacific and SE Atlantic (and SW Pacific and N Atlantic, Supplementary Fig. 3), and coincides with persistently low abundance of the extinction group thereafter. Vertical yellow and dashed lines as in Fig. 1.

Considering the strong benthic–pelagic coupling of benthic foraminifera^10^, we compiled published high-resolution calcareous nannoplankton assemblage records to assess the potential for a global changing source of organic carbon causing the extinction. Coccolithophores are one of the major mid-low latitude phytoplankton groups contributing to the organic carbon pump, known to undergo rapid evolution in the Pleistocene^25^. From published records, we identified that a significant peak in the morphological genus ‘small *Gephyrocapsa*' (<3 μm) occurred in the SE Atlantic^26^, NW Pacific^27^ and SW Pacific^11^ centred at ∼0.8 Ma (Fig. 3b). This peak in abundance is consistent with records from the Indian Ocean^25^, and other nannoplankton records that do not differentiate this particular species from other small placoliths in the North Atlantic, South Atlantic and Mediterranean Sea (Supplementary Fig. 2). Although most of these sites do not have benthic foraminiferal data with which to directly compare the nannoplankton assemblages, Site MD97–2114 in the SW Pacific indicates that the benthic extinction coincided with this peak^11^, and other sites^7^ indicate that at a global scale the extinction had largely taken place by 0.8 Ma (Fig. 3c). To test the hypothesis that dominant small *Gephyrocapsa* could have been implicated in the benthic extinction, we paired records of nannoplankton and foraminiferal assemblage data in the Tasman Sea (Fig. 1c) and the North Atlantic (Supplementary Fig. 3). Both records show a consistently high % small *Gephyrocapsa* (>90%) during the extinction interval at ∼0.83 Ma. Interestingly, the first step in the extinction at ∼0.96 Ma at Site 593 (Fig. 1g) coincides with an initial increase in the % small *Gephyrocapsa* from ∼50 to 65% (Fig. 1c), showing the possibility that % small *Gephyrocapsa* was already exerting some control over extinction group abundance. From our research, the abundance of small *Gephyrocapsa* is the only oceanic parameter that shows a correlation with the extinction group leading up to the extinction in the Tasman Sea (Fig. 2d). To address the possible significance of this correlation, we explore the various possible environmental changes controlling the two groups and describe a new conceptual model that can account for both data sets.

## Discussion

Our new nannoplankton data (Fig. 1c and Supplementary Fig. 3), together with other compiled records (Fig. 3b and Supplementary Fig. 2), indicate that the global oceans became dominated by small *Gephyrocapsa* around ∼0.8 Ma, a finding that has not previously been highlighted, perhaps because of the relatively short duration of this event compared with typical biostratigraphic sampling resolution. In some of the records, the dominance may have occurred sooner (for example, Site 980, N Atlantic) than others (for example, Site 593, Tasman Sea), such that there is diachroneity in the onset of this event; however, the crucial point is that all global records with coccolith data show high abundances of small *Gephyrocapsa* at ∼0.8 Ma. In summary, evidence for a global peak in small *Gephyrocapsa* at ∼0.8 Ma comes from sites in the SE Atlantic^26^, SW Pacific^11^, NW Pacific^27^, Indian Ocean^25^, N Atlantic and Tasman Sea (Supplementary Figs 2 and 3). The cause of the small *Gephyrocapsa* event remains unknown; however, it may have been an evolutionary adaptation^25^,^28^ as the *Gephyrocapsa* lineage is thought to be relatively independent of temperature and nutrients, but dependent on light intensity and day length^25^. The well-known abundance peak of *G. caribbeanica* at ∼0.6 Ma has been linked to increased blooms because of a sustained reduction in orbital eccentricity affecting day length and light intensity^25^; a sustained eccentricity minima is also observed during the benthic extinction event at ∼0.8 Ma (Supplementary Fig. 2).

**Figure 4:**
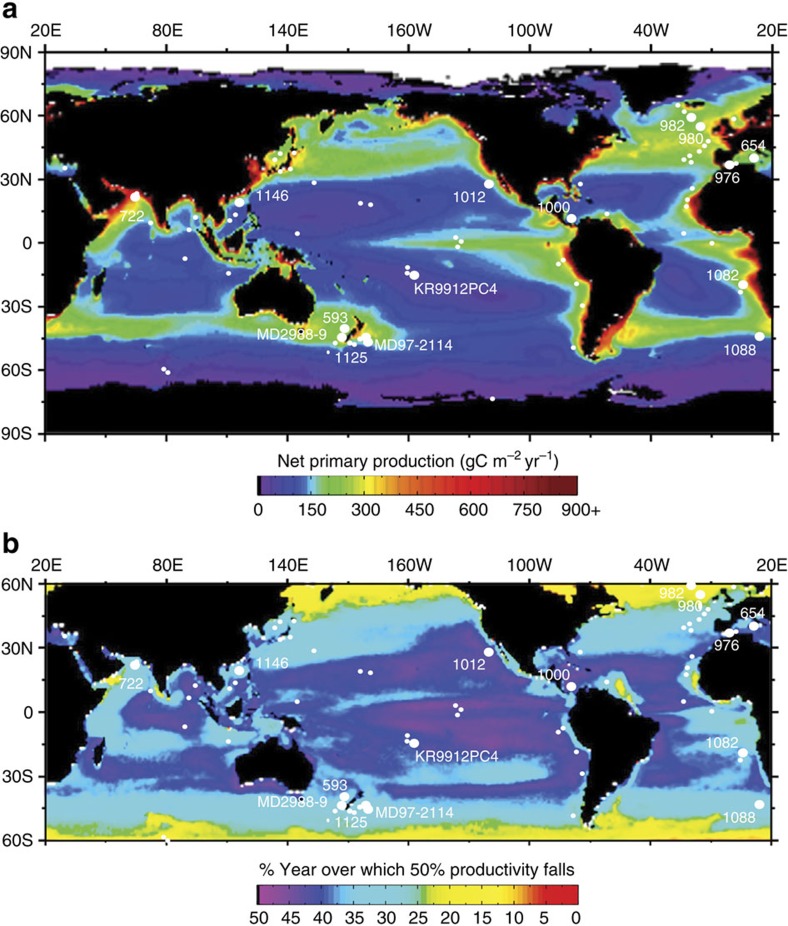
Distribution of extinction group study sites and modern productivity. Location of core sites on maps showing the mathematical estimates of modern net primary production, and modern unevenness of annual primary production (figure adapted from ref. 53). Sites shown indicate where the Mid Pleistocene benthic foraminiferal extinction group has been studied; the DSDP/ODP site numbers are given for higher-resolution studies^7^ that correspond to the sites used to construct Fig. 3. (**a**) Global average net primary productivity^53^. (**b**) The geographic distribution of the seasonal variation of net primary production (seasonality) for the years 1998–2007. The colours refer to the % of the year over which 50% productivity takes place, such that purple indicates no seasonality (colours modified from ref. 53). We propose that glacial MPT seasonality may have been higher than modern seasonality. Note that seasonality is generally lowest in regions where primary productivity is low.

Regardless of the cause of the small *Gephyrocapsa* event, its correlation with the extinction at ∼0.8 Ma provides a new piece of evidence when evaluating the cause of the benthic extinction. Correlation itself does not prove causation; however, as phytoplankton provide a food supply for marine benthos, how might this change in phytoplankton assemblages have had an impact on benthic foraminifera? We propose that the most plausible extinction mechanism would be a change towards a more enhanced variation in annual export production and delivery of organic carbon to the deep ocean^9^,^10^,^11^. There is micropalaeontological evidence for an increase in intra-annual variability of phytodetrital pulses during the extinction interval, with increased seasonal phytodetrital benthic foraminifera in the Indian Ocean^9^ and SW Pacific^11^ from ∼0.8 Ma. Previous studies have suggested that this could not have caused the benthic extinction because physical processes that enhance seasonality could not have occurred throughout the global ocean at the same time, as the benthic extinction did^7^,^11^. However, if the ecology of small *Gephyrocapsa* was similar to its modern descendent, *Emiliania huxleyi*, a seasonal bloom species that dominates mid-low latitude nannoplankton assemblages during the Holocene^25^,^28^, the increased global presence of small *Gephyrocapsa* may represent an early adaptation to more bloom-type ecology within nannoplankton, where intra-annual export production changed. Modern (interglacial) global productivity is dominantly annually uneven outside the oligotrophic ocean gyres (Fig. 4). Modelling studies indicate that mid-low latitude oceanic intra-annual variability of production may decrease with future projected warming^29^, potentially implying that intra-annual production variability could have been higher (compared with modern variability) during colder glacials. Any increased intra-annual variability during MPT glacials would have reduced organic carbon flux during some seasons, while increasing flux at other times. In addition to preferring relatively high organic carbon flux^7^,^9^, the unique apertural ornamentation of the extinction group may denote a different feeding strategy. We speculate that they required a more uniform supply of carbon throughout the year. This is in contrast to extant species, typically able to survive for years without fresh phytodetritus^30^. The phytoplankton change may have primed the group for complete extinction at ∼0.8–0.6 Ma. There remains the possibility that both the small *Gephyrocapsa* event and the benthic extinction reflect an alternative, common cause. Although we consider it unlikely, as we have discounted various water property changes (Figs 1 and 2), perhaps an unconstrained factor such as a reduction in surface and deep-ocean CO_2_ played a role.

Our proposed mechanism is consistent with the timing, bathymetric and geographic distribution of the extinction event. To summarize, our proposed ecology for the extinction group is that they preferred a relatively high flux of organic carbon *regularly* delivered to the sea floor such that there were no long periods within the year where flux was very low. This explains their distribution before the extinction^7^, which was higher in mid-low latitude regions as opposed to high latitudes, as high latitudes would have had a more uneven supply of export production throughout the year (Fig. 4). It was also lower in oligotrophic regions such as the South Pacific Gyre, South China Sea and Mediterranean Sea, and higher in modern eutrophic regions^7^. Finally, it was higher at mid-bathyal to upper-abyssal depths compared with the oligotrophic lower abyssal ocean, where the carbon flux is very low. This proposed ecology also explains their disappearance first at deeper abyssal depths (oligotrophic), the Mediterranean (oligotrophic) and during glacials^7^, which may have experienced more variability of seasonal export production, according to modelling studies^29^. Our proposed mechanism can explain the extinction because we invoke evolution within a phytoplankton group prevalent in the global oceans where the extinction group was most abundant before the extinction. Thus, although small *Gephyrocapsa* may not have been abundant at higher latitudes where siliceous phytoplankton predominate today, this was a region with low extinction group abundance before 0.8 Ma and thus not a suitable refuge for the group when mid-low latitude small *Gephyrocapsa* began to dominate nannoplankton assemblages.

The long-term decline of the extinction group during the Eocene–Oligocene, and increase in phytodetritus-exploiting species, has been suggested to be because of enhanced seasonality in primary productivity forced by cooling^10^,^31^. In this sense, the Cenozoic decline of the extinction group could be categorized as a ‘slow' mass extinction^32^, as export production may have exhibited gradually more intra-annual variability. However, the abrupt decline by ∼0.8 Ma coinciding with the small *Gephyrocapsa* event would indicate a ‘rapid' mass extinction with biological causes, perhaps with similarities to planktonic foraminiferal mass extinctions during the Palaeocene–Eocene and Eocene–Oligocene transitions^32^. The timing of our proposed increase in this mid-low latitude intra-annual variability, inferred from the global small *Gephyrocapsa* dominance and benthic extinction, may be important for the development of climate during the MPT as variability of primary production could have had an impact on carbon export and storage^33^. Further modelling studies are needed to ascertain the impact of an enhanced intra-annual variability of production on climate (during MPT cooling), forced by evolutionary changes within the biosphere rather than physical processes alone.

## Methods

### DSDP Site 593 stratigraphy and age model

DSDP Site 593 (40°30.47′S, 167°40.47′E, 1,063 m water depth) was cored on the Challenger Plateau of the Tasman Sea in the SW Pacific Ocean. The upper 393 m of recovered sediment is foraminiferal-bearing nannofossil ooze, hosting very abundant and visually well-preserved benthic foraminifera^34^. A low-resolution, orbitally tuned stratigraphy was available to guide sampling based on a shipboard bio- and magnetostratigraphy and coarse-resolution benthic δ^18^O analyses on infaunal *Uvigerina* spp.^35^. The updated age model here has been generated after re-assigning this to the GTS2012 timescale^36^. Samples for the new isotope stratigraphy were analysed at 10–20-cm resolution in cores 593Z-1H-1 through 593Z-5H-2 (∼0–36.3 m depth) to yield a mean sample resolution of ∼5 kyr for the time period 0–1.1 Ma. The revised glacial–interglacial stratigraphy for the interval 0–0.4 Ma, based on *C. wuellerstorfi* oxygen isotopes and the Brunhes/Matuyama palaeomagnetic reversal, is presented in ref. 37. Here we created a revised isotope stratigraphy for the period 0.4–1.1 Ma using new δ^18^O analyses of the epifaunal species *C. wuellerstorfi* (Supplementary Fig. 4 and Supplementary Data 1). The tuning targets are shown in Supplementary Table 1. The Potaka tephra (1 Ma (ref. 38)) is clearly identified at 21.50 mbsf, and lies above a distinct benthic δ^18^O minimum, which is thus aligned to MIS 31. The top of the Olduvai magnetochron is not well represented, but the base of the Olduvai and the Gauss/Matuyama boundary were used to guide identification of the key isotope stages. A linear sedimentation rate is assumed between all tie points.

### Micropalaeontology analysis

Benthic foraminiferal analyses were carried out at the British Geological Survey, Keyworth, UK, and at the University of Leicester, UK, using an Olympus SZX10 binocular microscope. Seventy-eight sediment samples of ∼10 cc were oven dried at 40 °C and washed with deionized water over a 63-μm sieve to remove clays (at Durham University). The >63-μm fraction was then dried, and individual species of foraminifera were counted under a binocular microscope and transferred into cardboard reference slides. Residues were sieved into size fractions, and picked separately, according to a procedure outline in ref. 7, so that the >250-μm fraction had 100% picked, the >125-μm fraction had an average of 37% picked and the >63-μm fraction had an average of 21% picked (Supplementary Data 2). Fraction splits were used in order to increase the number of samples analysed because of the high volume of sediment. Abundance in splits was then normalized for dry sediment weight to estimate a total number of extinction group specimens per g of dried sediment for each sample^7^. A total of 31 species and taxonomic groups were identified using the taxonomic work of Hayward *et al.*^7^, and photographed using a Hitachi S-3600N scanning electron microscope in the Geology Department at the University of Leicester (Supplementary Fig. 5). The BFAR (benthic foraminiferal accumulation rate, specimens cm^2 ^kyr^−1^) was calculated by using sediment dry bulk density (*ρ*, in g cm^−3^), sedimentation rate (*ν*, in cm kyr^−1^) and number of specimens per g (*x*, specimens g^−1^) as follows:


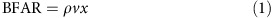


Calcareous nannofossil species were analysed at the British Geological Survey, Keyworth, UK. A total of 33 smear slides were prepared from the central part of fresh core sediment samples and were analysed using a Zeiss cross-polarizing light microscope at × 1,000 magnification. A counting phase was performed to obtain a qualitative and quantitative description of the assemblages based on percentage abundances of each recognized taxon (Supplementary Data 3). Nannofossil counts were performed in random visual fields on slides where the nannofossils were homogeneously distributed. At least 300 specimens >3 μm were counted per slide in a varying number of fields of view. Specimens <3 μm were included in a separate set of counts to quantify their dominance. As a result, two counts were obtained: the total assemblage and the subdominant one excluding specimens <3 μm.

Most of the species identified in this study belong to the family Noelaerhabdaceae and are recognized at generic and specific levels: *G. oceanica*, *G. caribbeanica*, *G. muellerae* recognized during this interval as *G. margereli*^39^, *G. omega* and small *Gephyrocapsa* (specimens <3 μm, mainly constituted of *G. aperta* and *G. ericsonii*). Other taxa are present within the assemblages recognized at generic, specific and subspecific levels. At the generic level, minor taxa are represented by the genres *Syracosphaera*, *Pontosphaera*, *Reticulofenestra* and *Rhabdosphaera*. At the specific level, minor taxa are represented by the species *Pseudoemiliania lacunosa*, *R. asanoi*, *Helicosphaera carteri*, *H. hyalina* and *Umbilicoshpaera sibogae.* Finally, *Coccolithus pelagicus* and *Calcidiscus leptoporus* were distinguished at subspecific and submorphotype levels on the basis of their coccolith length. Refs 40,41^40^,^41^ have documented the existence of two *C. pelagicus* subspecies: *C. pelagicus pelagicus*, the cold form, and *C. pelagicus braarudii*, the temperate and upwelling waters form^42^. Three *C. leptoporus* morphotypes have been distinguished: *C. leptoporus* type small (<5.5 μm (refs 41, 43)); *C. leptoporus* type medium (5.5–8 μm; (refs 41, 43)); and *C. leptoporus* type large (>8 μm (refs 41, 43)).

### Chlorin and alkenone analyses

Separate original samples were freeze-dried, homogenized, and alkenones and chlorins (diagenetic transformation products of chlorophyll)^24^,^44^ were extracted with an organic solvent mixture of dichloromethane/methanol (Supplementary Data 4), following the microwave-assisted protocol of ref. 45. The microwave temperature was increased from room temperature to 70 °C over 2 min, held at this temperature for 5 min and then allowed to cool. Extracts were subsequently centrifuged in test tubes, and the supernatant dried by rotary evaporation before being divided into two aliquots for analysis. All analyses were carried out in the Geography Department at Durham University. Chlorins were analysed by ultraviolet–visible spectrophotometry, quantified at the 410 and 665 nm wavelengths, and normalized for extracted sample weight in g (ref. 45). One aliquot was dissolved again in a known volume of acetone, and analysed using a dionex photodiode array detector coupled to a quaternary pump^45^. Absorbance across the 350–850-nm wavelength was recorded and quantified at 410 and 665 nm, which corresponds to the diagenetic transformation products of chlorophyll^24^,^44^. Samples were analysed in triplicate, and the means are reported here (Supplementary Data 4). The average s.d. within samples was 0.44 units (410 nm) and 0.08 units (665 nm).

Alkenones were isolated from the second aliquot of the lipid extract using silica column chromatography, eluting with *n-*hexane (for hydrocarbons), dichloromethane (for ketones) and methanol (for polar compounds). Alkenones were quantified by Thermo Scientific Trace 1310 gas chromatograph fitted with a flame ionization detector. Separation was achieved with a fused silica column (30 m × 0.25 mm inner diameter) coated with 0.25 μm of 5% phenyl methyl siloxane (HP-5MS) and using He as the carrier gas. After injection, the following oven temperature programme was used: 60–200 °C at 20 °C min^−1^, 200–320 °C at 6 °C min^−1^, and then held at 320 °C for 35 min. SST was calculated using the U^K^_37_′ index^46^ and the global mean annual SST calibration^47^.

### Oxygen isotope analysis

Approximately four individual tests of *C. wuellerstorfi* from the >250-μm fraction of each sample were analysed using an IsoPrime dual inlet mass spectrometer plus Multiprep device at the NERC Isotope Geosciences Laboratory. δ^18^O is reported as per mille (‰) deviations of the isotopic ratios (^18^O/^16^O), and is calculated on the V-PDB scale using an internal laboratory standard that is routinely calibrated against NBS-19 standards (Supplementary Data 1). Average analytical reproducibility for δ^18^O of the calcite standard is <0.1‰.

### Trace elemental analysis

Approximately 10 pristine individuals (with white calcite, no visible clay and no visible recrystallization) per sample of benthic foraminifera *U. peregrina* and *C. wuellerstorfi* were selected from the >250-μm fraction. Particular care was taken to select the correct *U. peregrina* and *C. wuellerstorfi* morphotypes, as *Planulina/Cibicidoides* have been shown to have significant species-specific elemental fractionation^48^,^49^. Benthic foraminifera were cleaned following the oxidative procedure of ref. 50, and analysed first for calcium concentration using inductively coupled plasma optical emission spectrometer (ICP-OES) (Varian Vista). *U. peregrina* were then re-analysed at 100 p.p.m. [Ca] for Mg/Ca by ICP-OES and the *C. wuellerstorfi* samples were re-analysed at 10 p.p.m. [Ca] by ICP-MS (Element) for B/Ca. All trace element sample preparation and analysis occurred at the Godwin Laboratory for Palaeoclimate Research, Cambridge University (Supplementary Data 5). All analysed tests appeared visually well preserved, and low Fe/Ca and Mn/Ca values recorded simultaneously by ICP-OES indicate that samples were effectively cleaned and were devoid of diagenetic effects. Estimations of past IWT (intermediate water temperature) were then generated using the most recently published calibration from the Mg/Ca ratio of *Uvigerina*^2^,^51^ (in mmol mol^−1^), which has been shown to be suitable for this site for the interval from 0.4–0 Ma (ref. 37).


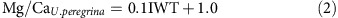


Bottom-water Δ[CO_3_^2−^] (defined as the difference between [CO_3_^2−^]_*in situ*_ and [CO_3_^2−^]_saturation_) was calculated from the B/Ca ratio of epifaunal foraminifera *C. wuellerstorfi* (in μmol mol^−1^) using the following calibration^49^. This proxy has also previously been used at this site for the interval from 0 to 0.4 Ma (ref. 37).


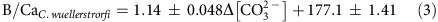


### Data availability

The data that support the findings of this study are available within the article and its Supplementary Information Files.

## Additional information

**How to cite this article:** Kender, S. *et al.* Mid Pleistocene foraminiferal mass extinction coupled with phytoplankton evolution. *Nat. Commun.* 7:11970 doi: 10.1038/ncomms11970 (2016).

## Supplementary Material

Supplementary Information Supplementary Figures 1-5, Supplementary Table 1 and Supplementary References.

Supplementary Data 1 Cibicidoides wuellerstorfi oxygen isotope data between 0.4 and 1.1 Ma for DSDP Site 593.

Supplementary Data 2 Foraminiferal census data for DSDP Site 593.

Supplementary Data 3 Calcareous nannofossil census data for DSDP Site 593 and ODP Site 980.

Supplementary Data 4 Sediment UK37' and chlorin data for DSDP Site 593.

Supplementary Data 5 Benthic foraminiferal trace element data for DSDP Site 593.
