# Supplementary material for: Mid Pleistocene foraminiferal mass extinction coupled with phytoplankton evolution
Source: Nat Commun. 2016 Jun 17;7:11970. doi: 10.1038/ncomms11970 (PMC4915025; doi:10.1038/ncomms11970)
Supplement: Supplementary Information — Supplementary Figures 1-5, Supplementary Table 1 and Supplementary References. [file ncomms11970-s1.pdf]

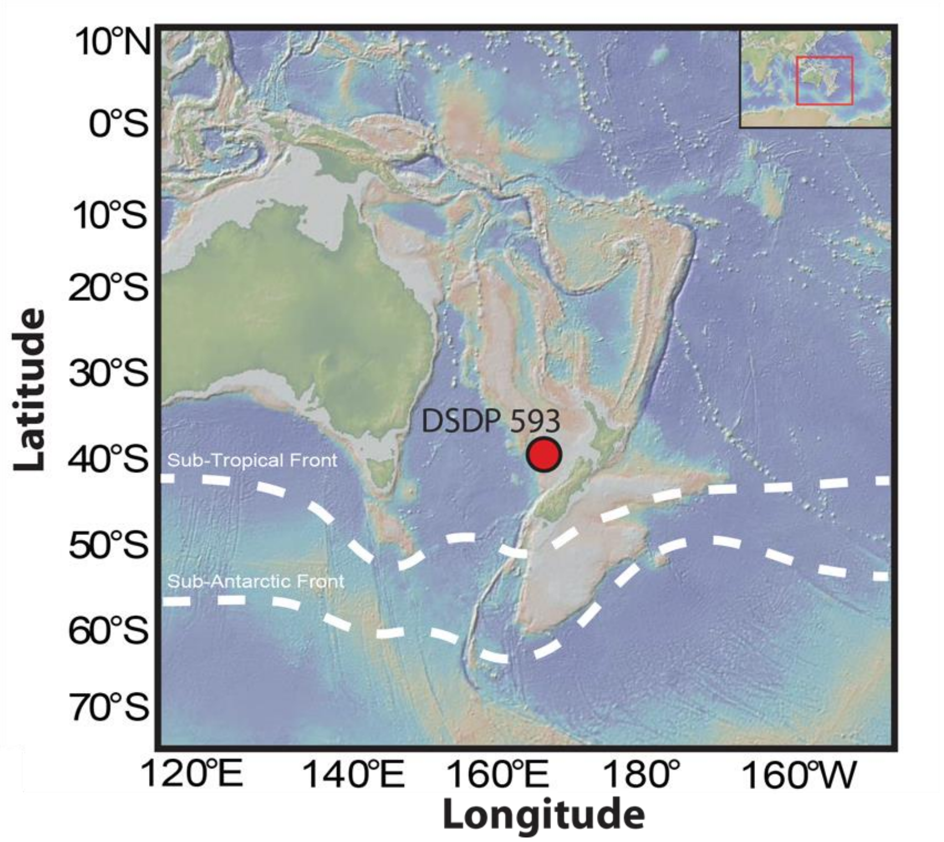

Supplementary Figure 1. Location bathymetry map of DSDP Site 593 (this study) in the Tasman Sea. Position of important frontal regions is also shown. Antarctic Intermediate Water, that currently bathes Site 593, forms between the two fronts. Map adapted from Elmore et al. (ref. 1).

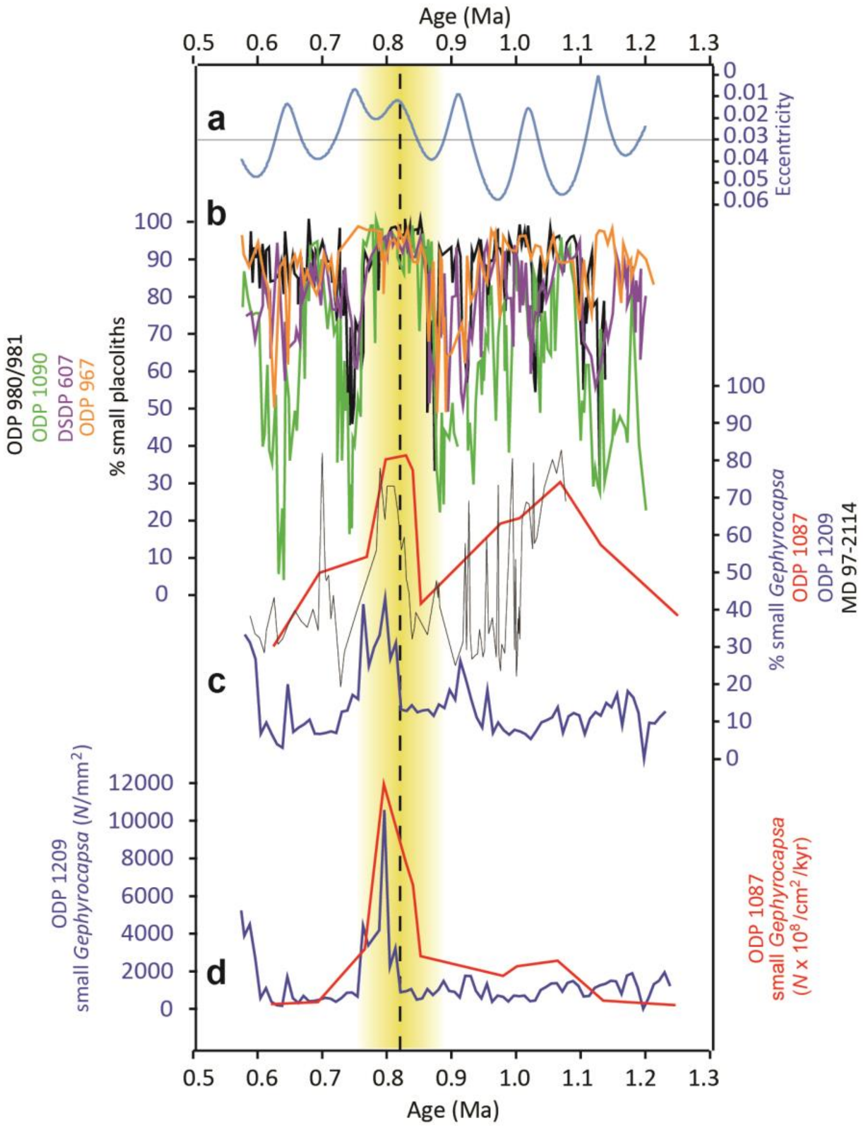

Supplementary Figure 2. Summary of previously-published Mid Pleistocene nannoplankton records. a, Orbital eccentricity[2], showing prolonged insolation minima at ~0.8 Ma due to minimum tilt. **b**, assemblage % small placoliths (which includes small *Gephyrocapsa*, along with other small species) from various sites[3,4,5]. **c**, % of small *Gephyrocapsa* from Sites 1087 (South Atlantic[6]), MD 97-2114 (SW Pacific[7]) and 1209 (North Pacific[8]). Yellow bar indicates the approximate position of the diachronous global benthic foraminiferal extinction (as in Fig. 1).

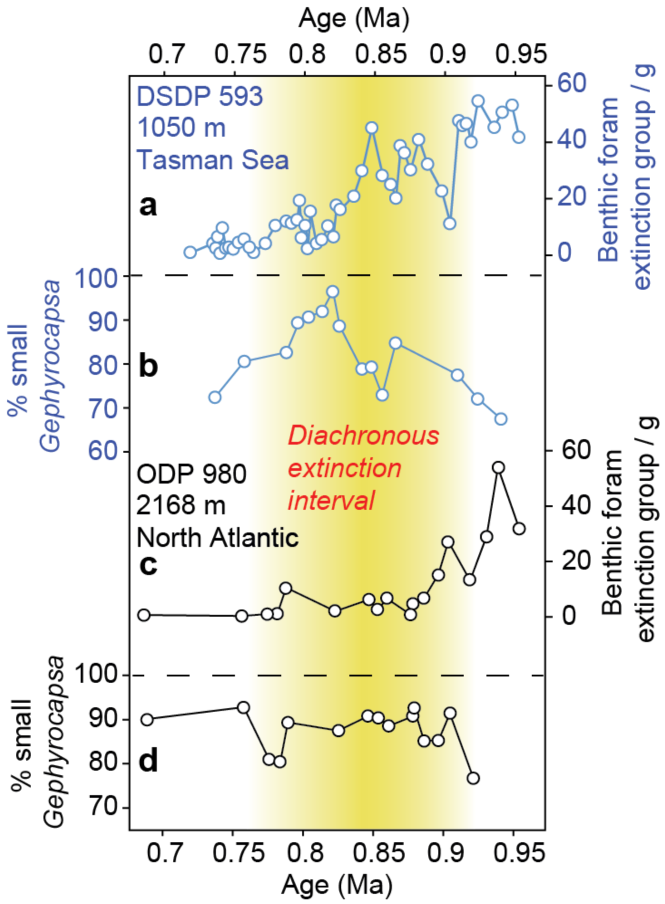

Supplementary Figure 3. Nannoplankton and extinct benthic foraminiferal assemblages compared at the same sites. a-b, DSDP Site 593 (this study). **c**, ODP Site 980 (ref. 9). **d**, ODP Site 980 (this study). Note how dominance of small *Gephyrocapsa* within nannoplankton is coincident with low abundance of the benthic foraminifera extinction group in both ocean basins in distal locations. Yellow bar indicates the approximate position of the diachronous global benthic foraminiferal extinction.

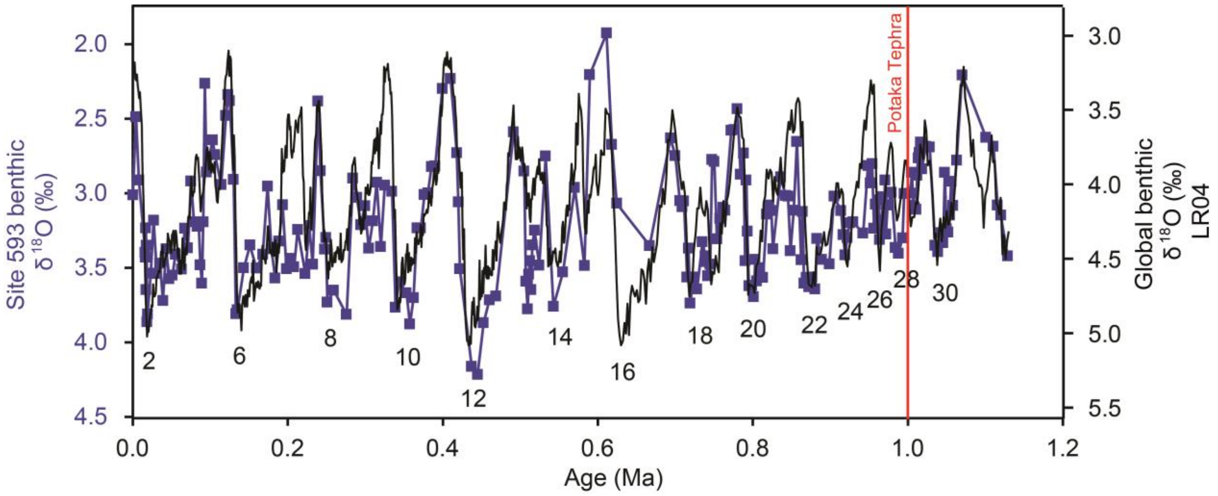

Supplementary Figure 4. Age model for DSDP Site 593. Plots show the correspondence between $\delta^{18}O_{C.\ wuellerstorfi}$ from DSDP Site 593 (blue; left axis; this study) with the LR04[10] benthic foraminiferal $\delta^{18}O$ global stack (black; right axis). Average analytical reproducibility for $\delta^{18}O$ of the calcite standard is <0.1‰. Numbers refer to isotope stages.

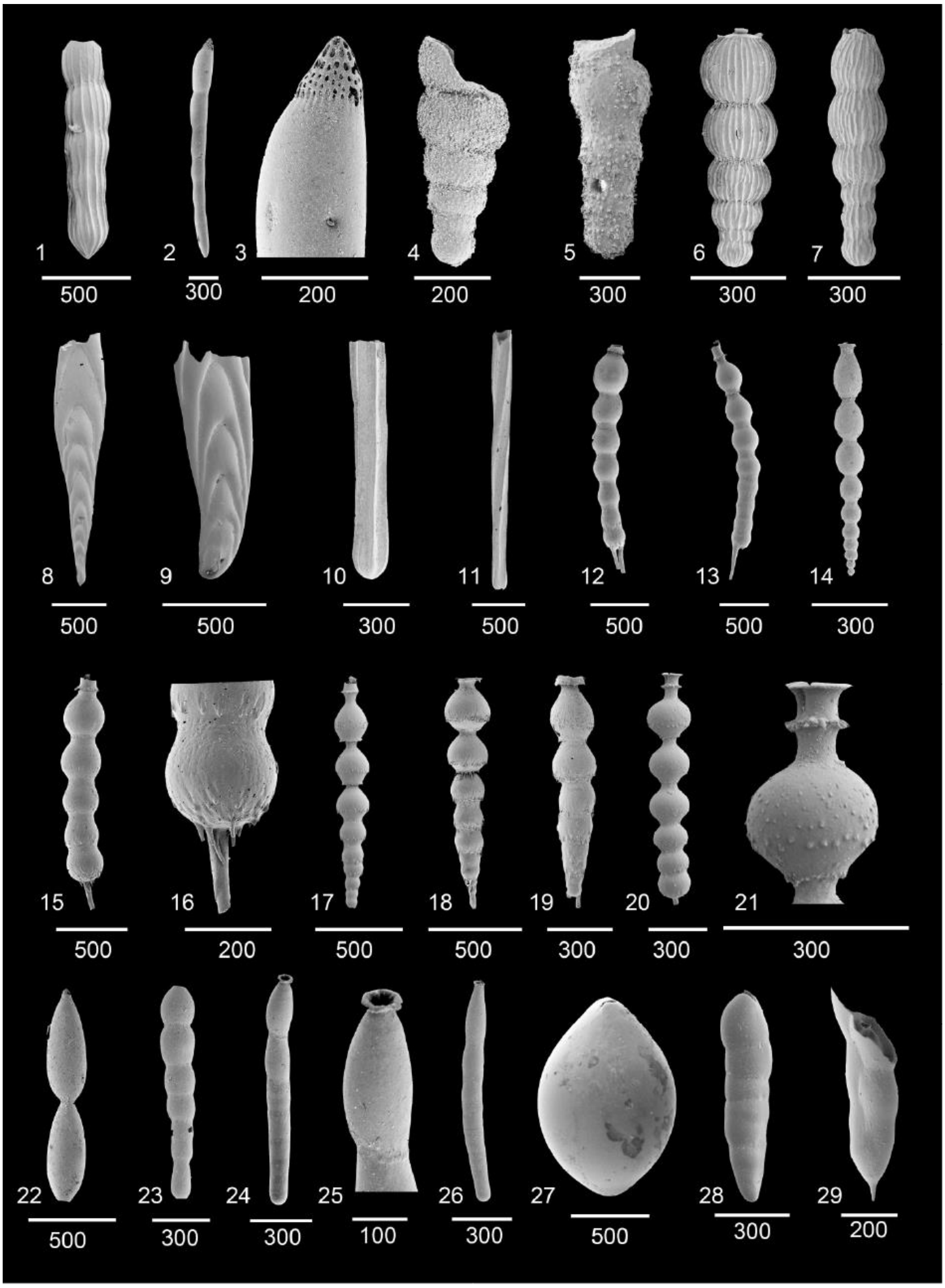

Supplementary Figure 5. Scanning electron microscope photographs of selected abundant taxa from the elongate uniserial Extinction Group. 1. *Chrysalogonium deceptorum* (593Z, 3H, 2W, 130-132 cm); **2-3.** *Cribroconica stimulea* (593Z, 3H, 4W, 100-102 cm); **4.** *Chrysalogonium rudis* (593Z, 3H, 6W, 40-42 cm); **5.** *Chrysalogonium rudis*

(593Z, 3H, 6W, 40-42 cm); **6.** *Orthomorphina perversa* (593Z, 3H, 4W, 100-102 cm); **7.** *Orthomorphina perversa* (593Z, 3H, 2W, 130-132 cm); **8.** *Mucronina compressa* (593Z, 3H, 2W, 130-132 cm); **9.** *Mucronina compressa* (593Z, 3H, 2W, 130-132 cm); **10.** *Staffia tostata* (593Z, 3H, 6W, 40-42 cm); **11.** *Staffia tostata* (593Z, 3H, 6W, 40-42 cm); **12.** *Siphonodosaria pomuligera* (593Z, 3H, 4W, 100-102 cm); **13.** *Siphonodosaria pomuligera* (593Z, 3H, 4W, 100-102 cm); **14.** *Strictocostella matanzana* (593Z, 3H, 6W, 40-42 cm); **15-16.** *Siphonodosaria jacksonensis* (593Z, 3H, 4W, 100-102 cm); **17.** *Siphonodosaria lepidula* (593Z, 3H, 2W, 130-132 cm); **18.** *Siphonodosaria lepidula* (593Z, 3H, 4W, 100-102 cm); **19.** *Siphonodosaria lepidula* (593Z, 3H, 6W, 40-42 cm); **20-21.** *Siphonodosaria lepidula* (593Z, 3H, 2W, 130-132 cm); **22.** *Stilostomella fistuca* (593Z, 3H, 4W, 100-102 cm); **23.** *Stilostomella parexilis* (593Z, 3H, 6W, 40-42 cm); **24-25.** *Strictocostella scharbergana* (593Z, 3H, 4W, 100-102 cm); **26.** *Strictocostella scharbergana* (593Z, 3H, 6W, 40-42 cm); **27.** *Ellipsoglandulina labiate* (593Z, 3H, 4W, 100-102 cm); **28.** *Pleurostomella alternans* (593Z, 3H, 2W, 130-132 cm); **29.** *Pleurostomella alternans* (593Z, 3H, 2W, 130-132 cm). Scale bars in µm.

Supplementary Table 1. Age/depth tie points used to construct the age model between 0.4 and 1.1 Ma for DSDP Site 593. These are based on visual comparison to the LR04 benthic foraminiferal $\delta^{18}O$ stack[10] unless noted above, see Supplementary Fig. 4.

Core Depth (m)	Age (Ma)	Reference
0.00	0	Ref. 1
0.31	0.0159	AMS-^{14}C (refs. 11,12)
0.81	0.088	Ref. 1
1.80	0.123	Ref. 1
2.31	0.138	Ref. 1
3.18	0.186	Ref. 1
3.86	0.237	Ref. 1
4.89	0.252	Ref. 1
5.28	0.295	Ref. 1
5.60	0.332	Ref. 1
5.80	0.341	Ref. 1
7.61	0.370	Ref. 1
8.07	0.421	Ref. 1
9.81	0.491	*This study*
10.31	0.513	*This study*
10.51	0.530	*This study*
11.01	0.584	*This study*
11.12	0.600	*This study*
12.00	0.650	*This study*
12.26	0.695	*This study*
12.81	0.706	*This study*
14.90	0.718	*This study*
15.10	0.735	*This study*
15.67	0.766	*This study*
15.88	0.790	*This study*
16.80	0.809	*This study*
17.17	0.831	*This study*
17.70	0.858	*This study*
18.10	0.874	*This study*
18.35	0.907	*This study*
18.56	0.92	*This study*
19.59	0.954	*This study*
21.20	0.987	*This study*
21.50	1.000	Potaka Tephra *(This study)*
23.50	1.070	Base of Jaramillo (ref. 13)
25.22	1.128	*This study*

Supplementary References

1. Elmore. A. C. *et al.* Antarctic Intermediate Water properties since 400ka recorded in infaunal (*Uvigerina peregrina*) and epifaunal (*Planulina wuellerstorfi*) benthic foraminifera. *Earth Planet. Sci. Lett.* **428**, 193–203 (2015).
2. Laskar, J. *et al.* A long-term numerical solution for the insolation quantities of the Earth. *Astron. Astrophys.* **428**, 261–285 (2004).
3. Marino, M., Maiorano, P. & Lirer, F. Changes in calcareous nannofossil assemblages during the Mid-Pleistocene Revolution. *Mar. Micropaleontol.* **69**, 70–90 (2008).
4. Marino, M., Maiorano, P., Lirer, F. & Pelosi, N. Response of calcareous nannofossil assemblages to paleoenvironmental changes through the mid-Pleistocene revolution at Site 1090 (Southern Ocean). *Palaeogeogr. Palaeoclimatol. Palaeoecol.* **280**, 333–349 (2009).
5. Marino, M., Maiorano, P. & Flower, B. P. Calcareous nannofossil changes during the Mid-Pleistocene Revolution: Paleoecologic and paleoceanographic evidence from North Atlantic Site 980/981. *Palaeogeogr. Palaeoclimatol. Palaeoecol.* **306**, 58–69 (2011).
6. McClymont, E. L. *et al.* Alkenone and coccolith records of the mid-Pleistocene in the south-east Atlantic: Implications for the U^{K}_{37}' index and South African climate. *Quat. Sci. Rev.* **24**, 1559–1572 (2005).
7. Mancin, N., Hayward, B. W., Trattenero, I., Cobianchi, M. & Lupi, C. Can the morphology of deep-sea benthic foraminifera reveal what caused their extinction during the mid-Pleistocene Climate Transition? *Mar. Micropaleontol.* **104**, 53–70 (2013).
8. Lupi, C., Bordiga, M. & Cobianchi, M. *Gephyrocapsa* occurrence during the Middle Pleistocene transition in the Northern Pacific Ocean (Shatsky Rise). *Geobios* **45**, 209–217 (2012).

9. Hayward, B. W. *et al.* The last global extinction (Mid-Pleistocene) of deep-sea benthic foraminifera, *Cushman Foundation for Foraminiferal Research Spec. Publ*. **408** (2012).

10. Lisiecki, L. E. & Raymo, M. E. A Pliocene-Pleistocene stack of 57 globally distributed benthic $\delta^{18}O$ records. *Paleoceanography* **20**, PA1003 (2005).

11. Nelson, C. S. in *Initial Reports DSDP* **90** (eds Kennett, J. P. *et al.*) 1471–1491 (Washington, U.S. Government Printing Office, 1986).

12. Dudley, W. C. & Nelson, C. S. Quaternary surface-water stable isotope signal from calcareous nannofossils at DSDP Site 593, Southern Tasman Sea. *Mar. Micropaleontol*. **13**, 353–373 (1989).

13. Cooke, P. J., Nelson, C. S. & Crundwell, M. P. Miocene isotope zones, paleotemperatures, and carbon maxima events at intermediate water-depth, Site 593, Southwest Pacific. *New Zeal. J. of Geol. Geop.* **51**, 1–22 (2008).
